# Dose-Response Effect of Oral Caffeine Use on Aerobic Exercise Performance: A Systematic Review and Meta-Analysis

**DOI:** 10.3390/nu18121989

**Published:** 2026-06-19

**Authors:** Gabriel L. Martins, Juliana M. Aparecido, Marcelo L. Marquezi, Caroline S. Frientes, Leonardo R. Miedes, Matheus S. Fornel, Tiago Fernandes, Antônio Herbert Lancha

**Affiliations:** 1 Plenitude Education Institute, Sao Paulo 01409-000, SP, Brazil; 2 Laboratory of Applied Nutrition and Metabolism, School of Physical Education and Sport, University of Sao Paulo, Sao Paulo 05508-030, SP, Brazil; 3 Laboratory of Physical Education Research (LAPEF), University City of Sao Paulo (UNICID), Sao Paulo 03071-000, SP, Brazil; juliana.monique@unicid.edu.br (J.M.A.); mlmqzz@gmail.com (M.L.M.); caroline.frientes@gmail.com (C.S.F.); leomiedes@gmail.com (L.R.M.); 4 Paulista School of Nursing, Federal University of Sao Paulo, Sao Paulo 04024-002, SP, Brazil; mfornel@hotmail.com; 5 Laboratory of Biochemistry and Molecular Biology of Exercise, School of Physical Education and Sport, University of Sao Paulo, Sao Paulo 05508-030, SP, Brazil; 6 Laboratory of Clinical Investigation 26: Experimental Surgery, Department of Surgery, Hospital das Clínicas, Medical School, University of Sao Paulo, Sao Paulo 01246-903, SP, Brazil

**Keywords:** caffeine, aerobic exercise, moderate caffeine doses, low caffeine doses, ergogenic effect

## Abstract

**Background/Objective:** Caffeine has demonstrated ergogenic effects across various doses (2–9 mg·kg^−1^). However, aerobic responses to caffeine vary substantially, with time-trial performance ranging from ~–3% to +16%. Given that higher doses may increase adverse effects without clear additional benefits, this review examined the effects of low (≤3 mg·kg^−1^), moderate (4–6 mg·kg^−1^), and high (>6 mg·kg^−1^) caffeine doses on time-trial performance. **Methods:** A systematic review and meta-analysis of randomized, placebo-controlled trials was conducted using PubMed, Embase, and Virtual Health Library databases. Eligible studies included healthy adults (18–59 years) acutely ingesting oral anhydrous caffeine before aerobic time-trial tests, with performance outcomes measured exclusively as time-to-completion variables. Data were pooled using standardized mean differences (SMDs) and 95% confidence intervals under random-effects models, and risk of bias was assessed using the Cochrane Risk of Bias tool. **Results:** Forty-eight studies (689 participants) met the inclusion criteria. Both low and moderate caffeine doses significantly reduced time-trial completion time relative to placebo. Low doses produced a standardized mean difference of −0.27 (95% CI: −0.44 to −0.11; *p* = 0.001), whereas moderate doses resulted in an SMD of −0.52 (95% CI: −0.77 to −0.28; *p* < 0.0001). No studies evaluating high caffeine doses (>6 mg·kg^−1^) and reporting time-to-completion outcomes met the inclusion criteria. Subgroup analyses demonstrated similar ergogenic effects in both trained and highly trained individuals consuming moderate caffeine doses. **Conclusions:** This is the first meta-analysis specifically focused on aerobic time-trial performance to suggest that pre-exercise ingestion of low caffeine doses (1.3–3 mg·kg^−1^) may enhance endurance performance by reducing time-trial completion time. Notably, the use of moderate caffeine doses (4–6 mg·kg^−1^) appears to produce a more consistent ergogenic effect.

## 1. Introduction

The popularity of caffeine as an ergogenic aid is not a recent phenomenon. The stimulant is supported by a substantial scientific foundation demonstrating its benefits on exercise performance, with a relatively favorable safety profile in studies conducted prior to the 2000s CE—a body of evidence that prompted the World Anti-Doping Agency (WADA) to remove caffeine from its list of “prohibited substances” as of 1 January 2004 [1]. Currently, caffeine remains included in the WADA Monitoring Program due to its widespread use among competitive athletes and its recognized ergogenic potential. By the early 2000s, caffeine supplementation had already gained prominence in position stands, consensus statements, and conferences organized by leading authorities in the field, such as the International Society of Sports Nutrition (ISSN) and the International Olympic Committee (IOC) [2,3]. At that time, both organizations classified caffeine within a select group of substances capable of enhancing aerobic sports performance, primarily due to its effects on adenosine receptors in the central nervous system (CNS), which are strongly associated with reduced perception of exertion (i.e., physical discomfort) and parallel increases in alertness and vigor when doses ranging from 3 to 6 mg of caffeine per kilogram of body mass were ingested prior to aerobic exercise.

Incorporating nearly a decade of new research, updated ISSN and IOC position reinforce previous statements. Although there is substantial variability in performance responses following caffeine ingestion, the updated guidelines consolidated that lower caffeine doses (~2 mg·kg^−1^) may positively influence aerobic exercise performance, with no apparent additional benefits from ingesting ≥ 9 mg·kg^−1^ [4,5]. In part, these developments motivated researchers to investigate whether caffeine could exert ergogenic effects across a broader dosage range (2–9 mg·kg^−1^) [6,7,8]. This line of inquiry is based on the hypothesis that caffeine may produce optimized CNS-mediated effects at low doses (2–3 mg·kg^−1^), while increases in dosage may or may not be accompanied by additional peripheral physiological effects—such as elevated ionic calcium concentrations and enhanced muscle fiber contractile force via actin–calcium–myosin interactions [8,9].

Additionally, a comprehensive review of 21 meta-analyses conducted by Grgic et al. (2020) [10] identified substantial variability in the magnitude of caffeine’s ergogenic effects across investigations focusing on time trial and time to exhaustion performance. The authors reported that caffeine could yield small to moderate improvements in motor performance (Cohen’s d ranging from 0.22 to 0.68). Given the methodological differences among meta-analyses evaluating caffeine’s impact on aerobic performance, including exercise protocol type, performance metrics, timing of caffeine administration, supplementation vehicle or form, and even the year the meta-analysis was conducted, these findings should be interpreted cautiously. Within this context, few meta-analyses have explicitly examined the dose–response relationship between caffeine and aerobic performance [11,12]. More recently, a network meta-analysis also investigated the effects of different caffeine doses and administration methods on time-trial performance [13]. However, previous investigations frequently combined distinct performance outcomes, such as time-to-completion, indices of TT performance and mean power output, while also pooling different caffeine delivery methods (e.g., capsules, chewing gum, and mouth rinse) within the same analytical framework. In addition, the most recent meta-analysis excluded studies administering caffeine doses < 2 mg·kg^−1^ [13]. These methodological differences may impair the interpretation of the isolated dose–response effects of orally ingested anhydrous caffeine on ecologically valid time-trial completion performance outcomes. Moreover, the meta-analyses reported different “average effect sizes” for performance improvements associated with moderate caffeine doses, ranging from small to moderate [11,12,13]. These discrepancies limit interpretability and hinder a clear understanding of caffeine’s dose–response effects in endurance-based sports (e.g., running, cycling, swimming, Ironman events), where final rankings are closely determined by precise temporal outcomes.

Based on this context, the present meta-analysis aims to comparatively investigate the effects of low (≤3 mg·kg^−1^), moderate (4–6 mg·kg^−1^), and high (>6 mg·kg^−1^) caffeine doses on performance outcomes exclusively related to time-trial completion across various exercise protocols. Given that caffeine consumption among competitive cyclists occurs primarily through caffeinated coffee and pharmaceutical preparations [14], and considering that caffeinated beverages may vary in caffeine content by more than 50% depending on factors such as cultivation conditions, bean type, and preparation method [15,16,17], the present investigation restricted inclusion criteria to studies administering pharmacological doses of anhydrous caffeine via oral ingestion (capsules or aqueous solutions).

## 2. Materials and Methods

### 2.1. Search Strategy

The search was restricted to a pre-established cutoff date of July 2022 (articles published up to 1 July 2022) and conducted in accordance with the Preferred Reporting Items for Systematic Reviews and Meta-Analyses (PRISMA) guidelines—Prospero: CRD42022384198 [18]. Academic articles were identified through searches in the following electronic databases and libraries: the US National Library of Medicine (PubMed), the Virtual Health Library (VHL), and Embase.

The “PICo” framework (P = Population; I = Intervention; Co = Context) guided the development of the search strategy, which incorporated a combination of keywords and descriptors connected using the Boolean operators “OR” and “AND.” An integrated title-based search was conducted using the following terms: “caffeine effect” AND “aerobic performance,” OR “cross country performance,” OR “time trial performance,” OR “running performance,” OR “endurance performance,” AND “high dose,” AND “low dose,” AND “different doses.” All reference selection and organizational procedures were managed using the mobile application designed for systematic reviews, Rayyan (Qatar Computing Research Institute, Doha, Qatar) [19], which facilitated real-time information sharing and workflow coordination among all authors involved in the present study.

### 2.2. Study Selection and Exclusion Criteria

Five independent reviewers (G.L.M., J.M.A., C.S.F., L.R.M., and M.S.F.) participated in the selection and screening process based on an initial evaluation of the titles and abstracts imported into the mobile application for systematic reviews. Each clinical article identified during the search was randomly assigned to one of the reviewers for assessment. For studies classified as “eligible” by a reviewer, a second evaluation was performed by another independent reviewer. In cases of disagreement regarding eligibility between the first two reviewers, the article was subsequently evaluated by a third reviewer (physiologist, DSc. M.L.M.) to determine the final decision. After the initial screening, full-text articles were examined by all five independent reviewers to verify whether the selected studies indeed met the eligibility criteria based on their methodological design and reported outcomes. When a study fulfilled all inclusion criteria but did not provide complete time-based performance measures (e.g., group means and standard deviations), the corresponding authors were contacted via email or through ResearchGate (ResearchGate GmbH, Berlin, Germany) to request the missing data. If the required information could not be obtained, the study was excluded due to the impossibility of performing proper analyses.

The inclusion criteria for the studies in our analyses were as follows: (1) Subjects—healthy adult individuals (ages 18–59 years); (2) Intervention—studies that examined only the effects of prior oral supplementation with pharmacological dosages of caffeine (via capsules or through an aqueous solution) in time trial aerobic tests lasting at least 3 min—thus characterizing a major use of aerobic energy metabolism in the exercise performed [20]; (3) Comparators—included a placebo group as a control; (4) Outcome—the intervention measured the improvement in time trial aerobic performance only through units of time measurement (such as seconds and/or minutes); (5) Publication period—original articles published until July 2022.

The exclusion criteria were: (A) The published clinical trial was not written entirely in English; (B) The pharmacological dosage of caffeine was not adjusted for the total weight of the participants; (C) Caffeine treatment involved dose fractionation during and before the start of performance tests; (D) Pharmacological use of caffeine was employed in combination with other known or potential ergogenic compounds (such as: creatine, beta-alanine, sodium bicarbonate, L-citrulline, or nitrates); (E) Caffeine administration was performed via dietary sources (e.g., filtered coffee and energy drinks) or through alternative forms of supplementation (such as: chewing gum, mouthwash, or sprays); (F) High-intensity interval training protocols and/or graded tests to exhaustion were performed; (G) Caffeine use was performed in the context of prior (partial or total) sleep deprivation; (H) Improvement in aerobic performance was measured through total work performed and/or total distance covered; (I) Data that could be used in this meta-analysis could not be obtained (absence of mean and standard deviation in performance tests).

### 2.3. Risk of Bias Assessment

After the randomized studies were selected through the search strategy, the risk of bias for each included study was evaluated using the “Risk of Bias” tool, version 2.0 (RoB2) [21,22], following the guidelines of the Cochrane Collaboration. The Cochrane tool for randomized controlled trials assesses risk of bias across the following domains: selection bias, performance bias, attrition bias, reporting bias, detection bias, and other potential sources of bias. For each domain, the risk of bias was classified as (1) low risk of bias, (2) unclear risk of bias, or (3) high risk of bias. It is important to note that the scale was used as an indicator of scientific evidence rather than as an exclusionary criterion.

### 2.4. Statistical Analysis

Aerobic performance measures from eligible studies (means and standard deviations) were used to construct forest plots in Review Manager software (RevMan, version 5.4.1; The Cochrane Collaboration, London, UK). A continuous random-effects model, based on the inverse variance method, was applied to efficiently calculate the effect size associated with the administration of low (≤3 mg·kg^−1^), moderate (4–6 mg·kg^−1^), or high (>6 mg·kg^−1^) caffeine doses (treatment group) compared with the effect generated under placebo conditions (control group). After study selection, all studies included within the moderate-dose category administered caffeine doses ranging from 4 to 6 mg·kg^−1^, as no eligible investigations using doses between 3.1 and 3.9 mg·kg^−1^ were identified. Effect size (ES) distribution was considered heterogeneous if the chi-square test (*I*^2^) reached statistical significance at *p* < 0.05, with a 95% confidence interval (95% CI). Heterogeneity was evaluated using the *I*^2^ statistic, with values of <25%, ≥50%, and ≥75% interpreted as low, moderate, and high heterogeneity, respectively [22].

Qualitative publication bias was also assessed for each forest plot through the construction of funnel plots and Kendall’s tau, which examined the dispersion of the standardized mean difference of each study relative to its standard error and the 95% CI of the pooled sample. If any study appeared outside the 95% CI limits of the overall analysis, an additional complementary forest plot was generated without the respective study to confirm the presence of any detected effect (Supplementary Figure S3).

## 3. Results

### 3.1. Study Selection

Our initial search identified 6948 article titles, which were reduced to 3010 after the removal of duplicate records using automation tools in Rayyan (Qatar Computing Research Institute, Doha, Qatar), followed by a secondary manual verification. After screening titles and abstracts—and excluding studies that were letters, reviews, meta-analyses, or original articles that did not assess exercise performance and/or did not administer pharmacological doses of caffeine specifically adjusted to participant body mass (mg·kg^−1^)—a total of 212 studies were selected for full-text reading and methodological assessment. Articles with abstracts in English but full texts available only in other languages, as well as studies not accessible in full (by databases or ResearchGate), were excluded, resulting in 203 articles eligible for full-text evaluation by the reviewers. Of the 203 studies initially selected for full-text review, we excluded 38 studies in which aerobic performance was assessed to exhaustion (rather than through time-trial performance tests); 37 studies due to the use of divergent performance outcome metrics (distance, watts, power output, etc.); 28 studies because caffeine administration was combined with other known or potential ergogenic substances; 21 studies in which caffeine was delivered through alternative forms (aerosols, chewing gum, or mouth rinses); 18 studies because caffeine dosage was fractionated at different moments (before and during time-trial tests); 9 studies because the total duration of the time-trial tests was under 180 s; and 4 studies due to the absence of complete performance-time data (means and standard deviations), which remained unobtainable after attempts to contact the authors (by e-mail or ResearchGate). Finally, 1 study was excluded after full-text assessment because caffeine was tested under conditions of prior sleep restriction.

**Figure 1:**
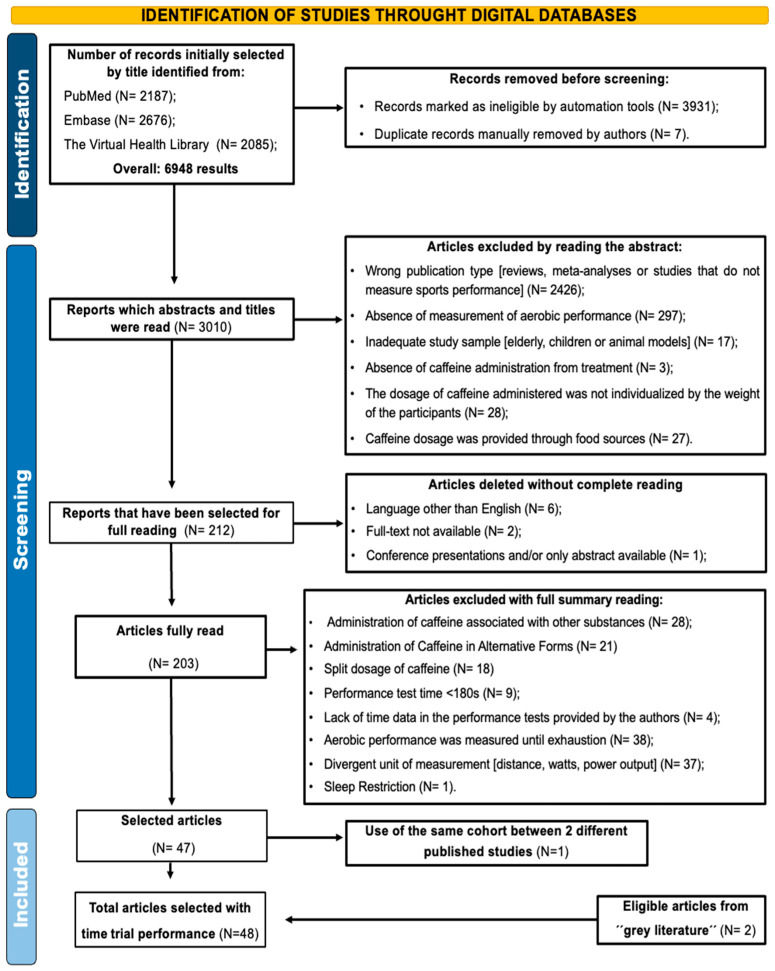
PRISMA flow diagram of research processes and excluded studies. Prepared from the PRISMA 2020 flow diagram [25].

After all exclusions, 47 articles remained. These studies were then subjected to a more detailed examination of their data, as well as to a verification of additional potentially eligible clinical trials cited within their reference lists (gray literature). During this process, it was identified that two of the selected articles [23,24] originated from the same cohort (registered under NCT 02109783), and therefore, one of them [23] was excluded to avoid duplicate analysis of the same group of individuals. Moreover, two additional eligible studies identified through gray literature sources were incorporated into this meta-analysis. In total, 48 studies were included in the present meta-analysis (Figure 1).

### 3.2. Study Characteristics

The characteristics of the eligible studies (N = 48) are summarized in a table (Table 1). Variables such as publication year, sex and number of participants, aerobic capacity (VO_2_max), acutely administered caffeine dose, timing of pre-exercise caffeine ingestion, the aerobic exercise protocol employed, as well as the mean change in performance observed in the caffeine-treated groups across the different pharmacological dosages (vs. placebo performance) were highlighted. The total sample consisted of 689 individuals (47 females [6.82%] and 642 males [93.18%]; mean participant age across studies ranged from 20 to 41.9 years). Ten studies (20.8%) did not report the cardiorespiratory fitness of their participants via VO_2_max or VO_2_peak. The pharmacological caffeine doses administered ranged from approximately 1.3 to 6 mg·kg^−1^ of body mass. Notably, no selected investigations employing high caffeine doses (>6 mg·kg^−1^) with performance outcomes quantified strictly by time (mean ± SD) were identified in the present assessment.

Regarding the characteristics of the time-trial performance tests included in the studies, 33 involved cycling (68.75%), 10 involved running (20.84%), 2 were rowing competitions (4.17%), 1 involved skiing (2.08%), 1 involved swimming competition (2.08%), and 1 was a triathlon event (2.08%). Finally, four studies [26,27,28,29] reported an ergolytic effect in the caffeine-treated groups relative to control (maximum performance decrement of −3%), whereas the greatest mean improvement in performance observed across the included studies was +15.9% compared with the placebo group.

**Table 1:** General characteristics of the studies included (N = 48).

Author/Year	Sample Size and Age (Years)	VO_2_max (mL/min/kg^−1^)	Caffeine Does (mg/kg^−1^)	Timing (min)	Exercise Protocol; DM	Change in Average Performance (Caffeine vs. Placebo)
Acker-Hewitt et al. (2012) [30]	10♂; 28 ± 9 years	66 ± 9	6	60	20 km cycling time trial; DM: 38.7 min	+1.35%
Al-Nawaiseh et al. (2020) [26]	11 (9♂ + 2♀); 24.5 ± 6.3 years	61 ± 6.1	5	60	5 km run; DM: ≈16.1 min	−2%
Astorino et al. (2011) [31]	16♂; 20.8–34 years	Physically active Group: 46.5 ± 6.3 Physically Trained Group: 57.5 ± 3.9	5	60	10 km Cycling time trial; DM: ≈16.1 min	Physically active Group: +0.96% Physically Trained Group: +1.61%
Astorino et al. (2012) [32]	10♀; 22.1 ± 1.9 years	* NR	6	60	8.2 km cycling time trial; DM: 16.7 min	+2.75%
Astorino et al. (2012) [33]	9 (8♂ + 1♀); 27.4 ± 5.9 years	57.5 ± 3.9	5	60	10 km Cycling time trial; DM: ≈16 min	+1.6%
Bell et al. (2002) [34]	12 (10♂ + 2♀); 33 ± 7 years	VO_2_peak: 57.5 ± 3.4	4	60	10 km run with an extra 11 kg load; DM: 43.2 min	+1.7%
Bloomer et al. (2011) [35]	12 (6♂ + 6♀); 21.9 ± 2.9 years	* NR	4	60	10 km run; DM: ≈50.1 min	+1%
Borba et al. (2019) [36]	13 (8♂ + 5♀); 18–40 years	* NR	6	60	1.6 km run; DM: 8.45 min	+0.39%
Bridge et al. (2006) [37]	8♂; 21.3 ± 1.2 years	* NR	3	60	8 km run; DM: ≈31 min	+1.2%
Conway et al. (2003) [38]	8♂; 25.5 ± 5 years	71.98 ± 3.9	6	60	Cycling time trial (work equivalent to 80% VO_2_max for 30 min); DM: 21 min	+15.9%
Couto et al. (2022) [39]	9♂; 32.3 ± 6 years	55.2 ± 5.7	5	60	4 km run; DM: ≈5.9 min	+3.17%
Cox et al. (2002) [40]	12♂; 27.1 ± 1.3 years	VO_2_peak: 66.4 ±1.3	6	60	Cycling time trial (Equivalent to 80% VO_2_max for 30 min); DM: ≈27.5 min	+ 3.4%
Dean et al. (2009) [41]	8♂; 36.4 ± 6.1 years	52.5 ± 6.1	3	60	40 km cycling time trial; DM: ≈58 min	+1.4%
Desbrow et al. (2009) [27]	9♂; 29.4 ± 4.5 years	VO_2_peak: 61.7 ± 4.8	1.5 and 3	60	Cycling time trial (Equivalent to approximately 82% PP for 30 min); DM: ≈26.3 min	1.5 mg·kg^−1^: −0.93% 3 mg·kg^−1^: +1.86%
Desbrow et al. (2012) [42]	16♂; 32.6 ± 8.3 years	VO_2_peak: 60.4 ± 4.1	3 and 6	90	Cycling time trial (Equivalent to approximately 75% PP for 1 h); DM: 57.53 min	3 mg·kg^−1^: +4.2% 6 mg·kg^−1^: +2.9%
Duncan et al. (2016) [43]	12♂; 24.6 ± 6 years	* NR	3	60	5 km run; DM: ≈21.5 min	+5.5%
Felippe et al. (2018) [44]	11♂; 34 ± 4 years	55 ± 4	5	60	4 km cycling time trial; DM: 6.5 min	+1.8%
Ferreira Viana et al. (2020) [45]	9♂; 32 ± 7.5 years	55 ± 6.1	6	60	4 km cycling time trial; DM: 5.6 min	+1.8%
Franco-Alvarenga et al. (2019) [46]	12♂; 34.3 ± 6.2 years	58.9 ± 6.2	5	50	20 km cycling time trial; DM: 31.2 min	+1.7%
Glaister et al. (2015) [47]	14♀; 31 ± 7 years	52.3 ± 4.9	5	60	20 km cycling time trial; DM: ≈33.4 min	+2.12%
Glaister et al. (2021) [48]	40♂; 41.9 ± 8.6 years	47.05–61.5	5	60	Cycling time trial (work equivalent to 85% Wmax for 25 min); DM: 27.9 min	+3.57%
Gonçalves et al. (2017) [49]	40♂; 37 ± 8 years	50.10 ± 8.45	6	60	Cycling time trial (work equivalent to 85% Wmax for 30 min); DM: ≈27.8 min	+2.89%
Graham-Paulson et al. (2016) [50]	11♂; 24 ± 4 years	VO_2_peak: 42.9 ± 7.3	4	45	10 km cycling time trial; DM: ≈22.8 min	+1.8%
Guest et al. (2020) [24]	100♂; 25 ± 4 years	VO_2_peak: 32–59	2 and 4	60	10 km cycling time trial; DM:17.4 min	2 mg·kg^−1^: +1.65% 4 mg·kg^−1^: +3%
Hanson et al. (2019) [28]	10 (6♂ + 4♀); 26 ± 9 years	46.9–71.6	3 and 6	60	10 km run; DM: 45.2 min	3 mg·kg^−1^: −0.38% 6 mg·kg^−1^: +0.94%
Hodgson et al. (2013) [51]	8♂; 25 ± 4 years	58 ± 3	5	60	Cycling time trial (Equivalent to 70% Wmax for 45 min); DM: ≈37.9 min	+4.27%
Irwin et al. (2011) [52]	12♂; 28.3 ± 5.8 years	VO_2_peak: 63.7 ± 7.4	3	90	Cycling time trial (work equivalent to 75% PP for 1 h); DM: 53.85 min	+2.5%
Khcharem et al. (2021) [53]	13♂; 21.3 ± 0.8 years	51.3 ± 6.1	3	60	3 km run; DM: 9.5 min	+1.1%
Kilding et al. (2012) [54]	10♂; 24.2 ± 5.4 years	* NR	3	60	3 km cycling time trial; DM: ≈3.62 min	+0.96%
Macintosh et al. (1995) [55]	11 (7♂ + 4♀); 22.9 ± 1.1 years	* NR	6	120–30	1.5 km swim test; DM: ≈20.4 min	+2.97%
Morales et al. (2020) [56]	14♂; 34.1 ± 4.4 years	51.5 ± 6.3	6	60	16 km cycling time trial; DM: ≈26.7 min	+ 2.55%
O’Rourke et al., (2008) [57]	30♂; 23.3–41 years	* NR	5	60	5 km run; DM: 16.3 min	Physically active Group: +1%; Physically Trained Group: +1.1%
Pitchford et al. (2014) [58]	9♂; 22–42 years	64.4 ± 6.8	3	90	Cycling time trial (Equivalent to 75% Wmax for 1 h); DM: 57.5 min	+6.7%
Pollow et al. (2016) [59]	7♂; 26.9 ± 3.9 years	VO_2_peak: 67.7 ± 10.3	6	60	50 km cycling time trial; DM: 80.9 min	+0.6%
Potgieter et al. (2018) [60]	26 (14♂ + 12♀); 37.8 ± 10.6 years	* NR	6	60	Triathlon (1.5 km swim, 40 km bike, 10 km run); DM: 129.8 min	+1.3%
Quinlivan et al. (2015) [61]	11♂; 31.7 ± 5.9 years	60.3 ± 7.8	3	90	40 km cycling time trial; DM: 58.50 min	+3.1%
Roelands et al. (2011) [29]	8♂; 23 ± 5 years	* NR	6	60	Cycling time trial (Equivalent to 75% Wmax for 30 min.); DM: 33.3 min	−3%
Santos et al. (2013) [62]	8♂; 32.6 ± 5.4 years	57.5 ± 5.8	5	60	4 km cycling time trial; DM: 6.6 min	+2.4%
Santos et al. (2020) [63]	16♂; 33.5 ± 5.2 years	High Performance Group: 57.3 ± 8.1 Low Performance Group: 48.9 ± 10	5	60	4 km cycling time trial; DM: 5.9 min	High Performance Group: +1.6% Low Performance Group: +2.5%
Silva-Cavalcante et al. (2013) [64]	7♂; 32.3 ± 5.4 years	VO_2_peak: 58.1 ± 6.3	5	60	4 km cycling time trial; DM: ≈6.5 min	+3.9%
Scott et al. (2015) [65]	13♂; 21 ± 2 years	39.6–52.8	1.3 ± 0.1	10	2 km rowing performance; DM: ≈7.3 min	+1.1%
Skinner et al. (2010) [66]	10♂; 20.6 ± 1.4 years	58.15 ± 6.8	2, 4, and 6	60	2 km rowing performance; DM: ≈6.4 min	2 mg·kg^−1^: +0.35% 4 mg·kg^−1^: +0.67% 6 mg·kg^−1^: +0.30%
Skinner et al. (2013) [67]	14♂; 31 ± 5 years	69.5 ± 6.1	6	60	40 km cycling time trial; DM: 56.3 min	+2%
Skinner et al. (2019) [68]	27 (16♂ + 11♀); 32.6 ±8.3 years	Women’s VO_2_peak: 51.9 ± 7.2 Men’s—VO_2_peak: 60.4 ± 4.1	3	90	Cycling time trial (work equivalent to 75% Wmax for 60 min); DM: ≈57.5 min	Women’s: +2.75% Men’s: +4.33%
Spence et al. (2013) [69]	10♂; 30 ± 2 years	VO_2_peak: 58.9 ± 2	2.5 ± 0.1	60	40 km cycling time trial; DM: ≈71.5 min	+1.29%
Stadheim et al. (2013) [70]	10♂; 20 ± 1 years	69.3 ± 1	6	75	8 km cross-country Double Poling; DM: ≈31.6 min	+3.64%
Tomazini et al. (2022) [71]	11♂; 33 ± 7 years	56.1 ± 13.2	5	60	4 km cycling time trial; DM: 6.4 min	+1.05%
Walker et al. (2008) [72]	9♂; 23 ± 3 years	71.2 ± 6.8	6	60	Cycling time trial (work equivalent to 70% PP for 30 min); DM: 25.4 min	+3.9%

CAF = caffeine group; PLA = placebo group; DM = Minimum duration of exercise performed among participants; * NR = Not Reported.

### 3.3. Risk of Bias Assessment and Funnel Plots

**Figure 2:**
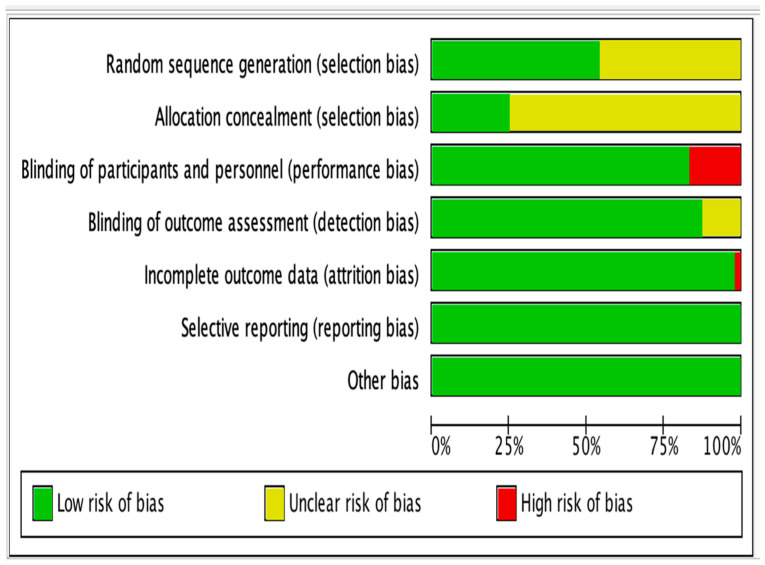
Global analysis by the authors on the risk of bias in studies that analyzed the influence of caffeine on aerobic performance. The analysis was performed using the Cochrane Risk of Bias analysis tool, version 2.0. The graph was created using the Review Manager 5.4.1 program, in its free version.

Risk of bias assessment was conducted for the 48 placebo-controlled crossover trials included in this review (Figure 2) and to observe the individual assessment of each study in Supplementary Figure S1. Eleven trials (22.92%) were classified as having a low risk of bias, with clear descriptions of the methodological domains evaluated—selection bias, performance bias, attrition bias, reporting bias, detection bias, and other domains. In contrast, 37 studies (77.08%) were classified as having an unclear risk of bias due to insufficient detail regarding randomization procedures and/or allocation of participants. Eight studies (16.6%) were identified as having a high risk of bias related to blinding procedures, either because assessor blinding was not implemented (single-blind methodological design) [28,31,51,65,72] or because blinding was compromised in more than 50% of the sample (i.e., despite the double-blind design, over half of the participants correctly identified whether they had ingested caffeine or placebo) [59,68].

Regarding outcome assessment, 42 studies (87%) were classified as having a low risk of bias, while only 6 were considered to have an unclear risk of bias. In the domain of data analysis, a single study (2.08%) was categorized as having a high risk of bias due to reporting participant attrition exceeding 20% of the initially described sample [30]. Finally, all studies evaluating time-trial performance (N = 48; 100%) were classified as having a low risk of bias for the domains of “selective reporting” and “other sources of bias.”

**Figure 3:**
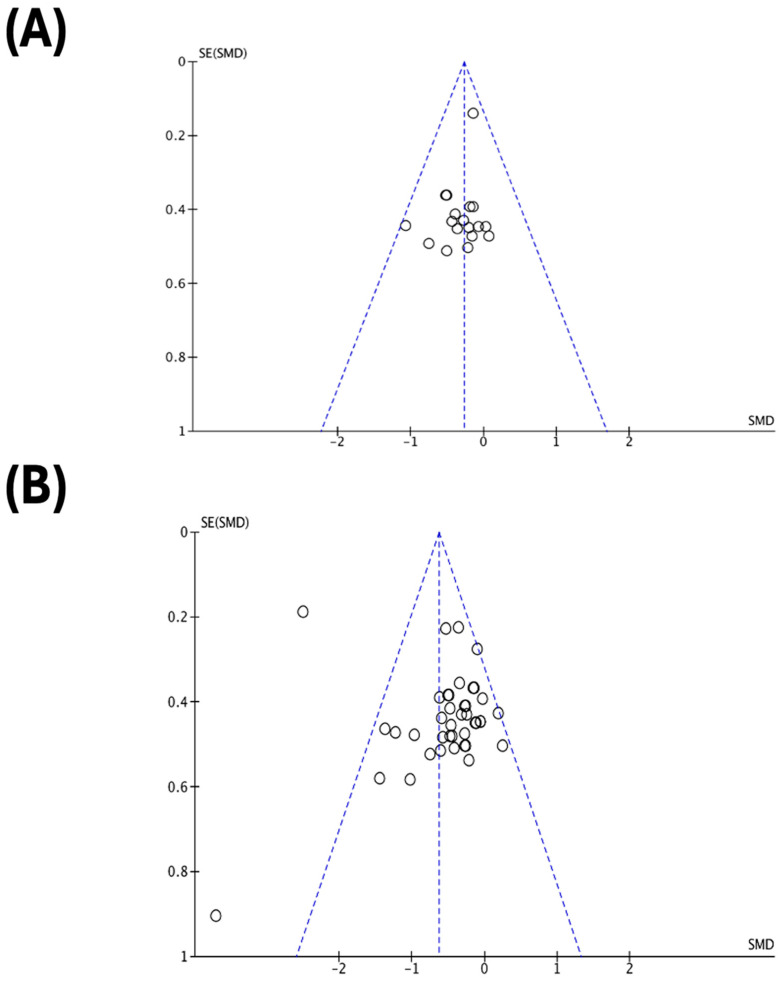
Funnel plot of studies comparing the use of caffeine treatment vs. control treatment (placebo) in aerobic performance tests. (**A**) Funnel plot of studies using low caffeine dosages (≤3 mg·kg^−1^) (**B**) Funnel plot of studies investigating the effects of moderate caffeine dosages (4–6 mg·kg^−1^) on time trial performance. Results from each of the analyzed studies are represented by circles, with the “y” axis representing the standard error of the data from each study and the “x” axis representing the difference from the standardized mean of their results. The graph was created using the Review Manager 5.4.1 program in its free version. The graph scale was represented as 4.5 SMD.

Analysis of the funnel plots for studies investigating low caffeine doses (N = 17; Figure 3A) and moderate caffeine doses (N = 36; Figure 3B) revealed that only two studies [24,51] fell outside the 95% confidence interval limits, both located on the left side of the funnel plot for moderate caffeine doses. Specifically, Hodgson et al. (2013) [51] employed a single-blind design, whereas Guest et al. (2020) [24] included a substantially larger sample size compared to most eligible studies and a low proportion of participants with the CYP1A2 CC genotype, commonly associated with slower caffeine metabolism and a potentially lower ergogenic response to caffeine. This sample profile may have contributed to the larger performance effect observed in this study and, consequently, to its dispersion outside the 95% confidence interval limits in the funnel plot analysis. Collectively, these findings suggest that study-specific methodological and participant characteristics may have contributed to the observed funnel plot asymmetry.

### 3.4. Meta-Analyses: Effect of Different Caffeine Dosages on Aerobic Time-Trial Performance

**Figure 4:**
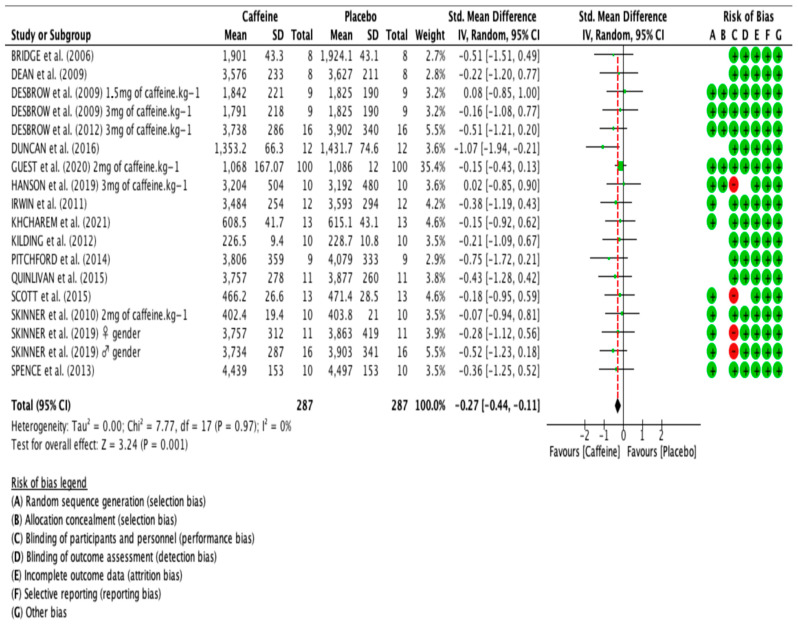
Forest plot for the effect of interventions using low doses of caffeine (~1.3 to 3 mg·kg^−1^) vs. the control group (placebo) on aerobic time trial performance tests. The analysis of the effects of the data was performed randomly, with the overall mean effect and respective standard deviation represented by a 95% CI. The chi-square (*I*^2^) percentage value represents the percentage of heterogeneity among the samples of the studies included in this meta-analysis. All time measurements computed in this meta-analysis were parameterized in seconds, with the mean performance time values for each treatment condition placed in the “Mean” column and their respective standard deviations in the “SD” column. The forest plot scale was set to 3.99 for better comparison with other analyses. The symbols displayed in the figure correspond to the standard graphical output generated by meta-analysis software. Individual studies are represented by circles, with larger circles reflecting studies with greater statistical weight (typically due to larger sample sizes and/or greater precision). The vertical dashed line represents the pooled effect estimate, while the diagonal lines represent the expected 95% confidence limits. Finally, the diamond at the bottom of the figure represents the overall pooled result of the meta-analysis, which constitutes the main outcome of the analysis. Studies included in this analysis: [24,27,28,37,41,42,43,52,53,54,58,61,65,66,68,69].

Sixteen clinical trials (33.3% of the studies) investigated the effects of low caffeine dosages (≤3 mg·kg^−1^) on time-trial performance, comprising a total of 287 participants in the caffeine-treated groups. The meta-analysis of these studies demonstrated that the ingestion of low caffeine doses (ranging from approximately 1.3 to 3 mg·kg^−1^) resulted in a significant improvement in total time to complete aerobic time-trial tests (SMD = −0.27, 95% CI = −0.44 to −0.11, *p* = 0.001, *I*^2^ = 0%)—For more details, see Figure 4.

**Figure 5:**
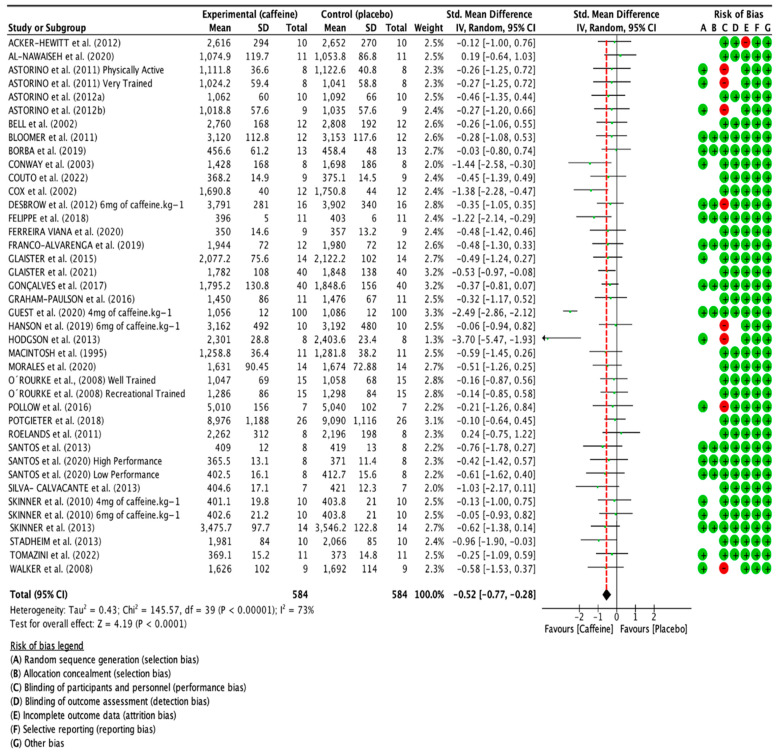
Forest plot for the effect of interventions using moderate doses of caffeine (4 to 6 mg·kg^−1^) and placebo control in aerobic time trial performance tests. The analysis of the effects of the data was performed randomly, with the overall mean effect and respective standard deviation represented by a 95% CI. The chi-square (*I*^2^) percentage value represents the percentage of heterogeneity among the samples of the studies included in this meta-analysis. All time measurements computed in this meta-analysis were parameterized in seconds, with the mean performance time values for each treatment condition placed in the “Mean” column and their respective standard deviations in the “SD” column. The forest plot scale was set to 3.99 for better comparison with other analyses. The symbols displayed in the figure correspond to the standard graphical output generated by meta-analysis software. Individual studies are represented by circles, with larger circles reflecting studies with greater statistical weight (typically due to larger sample sizes and/or greater precision). The vertical dashed line represents the pooled effect estimate, while the diagonal lines represent the expected 95% confidence limits. Finally, the diamond at the bottom of the figure represents the overall pooled result of the meta-analysis, which constitutes the main outcome of the analysis. Studies included in this analysis: [24,26,28,29,30,31,32,33,34,35,36,38,39,40,42,44,45,46,47,48,49,50,51,55,56,57,59,60,62,63,64,66,67,70,71,72].

Thirty-six eligible clinical trials (75% of the included studies) examined the effects of moderate caffeine dosages (4–6 mg·kg^−1^) on time-trial performance, comprising a total of 584 participants across the various caffeine treatment conditions. The meta-analysis of these studies demonstrated that the ingestion of moderate caffeine doses (ranging from 4 to 6 mg·kg^−1^) produced a significant improvement in total time to complete aerobic time-trial tests (SMD = −0.52, 95% CI = −0.77 to −0.28, *p* < 0.0001, *I*^2^ = 73%). Further details are presented in Figure 5.

### 3.5. Exploratory Analyses of Heterogeneity Among Studies Using Moderate Caffeine Doses

Due to the substantial heterogeneity observed in the primary meta-analysis involving moderate caffeine doses (*I*^2^ = 73%), additional exploratory subgroup and sensitivity analyses were performed to further investigate potential sources of variability among included trials.

Initially, subgroup analyses according to sex and caffeine ingestion timing were considered. However, these analyses were not performed because the currently available literature did not provide sufficiently balanced data distributions to allow meaningful subgroup comparisons. Specifically, only one eligible study reported performance outcomes separately by sex [68], whereas only one study was conducted exclusively in female participants [47]. Similarly, most eligible studies administered caffeine approximately 60 min before aerobic time-trial tests (N = 38), whereas only seven studies investigated longer ingestion intervals ranging from 75 to 120 min [42,52,55,58,61,68,70] and only three studies adopted shorter intervals ranging from 10 to 50 min [46,50,65]. Collectively, these findings indicate that the body of evidence currently available within the eligibility criteria of the present review does not provide a sufficient volume or distribution of data to support reliable subgroup analyses for these variables.

Considering the availability of VO_2_max/VO_2_peak data among a subset of included studies, an exploratory subgroup analysis according to aerobic training status was additionally performed among investigations administering moderate caffeine doses. Participants presenting VO_2_max/VO_2_peak values between ~40–60 mL·kg^−1^·min^−1^ were categorized as trained individuals, whereas participants with VO_2_max/VO_2_peak values above 60 mL·kg^−1^·min^−1^ were categorized as highly trained individuals, according to previously proposed aerobic fitness classifications [73,74]. Studies that did not report VO_2_max/VO_2_peak values, as well as studies including participants with substantial variability in aerobic fitness levels that precluded a clear classification of training status, were excluded from this exploratory subgroup analysis to improve the accuracy of participant categorization. This exploratory analysis demonstrated statistically significant ergogenic effects in both trained individuals (SMD = −0.81; 95% CI = −1.59 to −0.02; *p* = 0.04; *I*^2^ = 91%) and highly trained individuals (SMD = −0.94; 95% CI = −1.35 to −0.53; *p* < 0.00001; *I*^2^ = 0%), with no statistically significant subgroup differences observed between categories (*p* = 0.77). Further details are presented in Supplementary Figure S2.

## 4. Discussion

The purpose of this systematic review and meta-analysis was to evaluate the effects of low (≤3 mg·kg^−1^), moderate (4–6 mg·kg^−1^), and high (>6 mg·kg^−1^) caffeine doses on performance in aerobic-dominant time-trial events, such as long-distance running, cycling, swimming, and rowing. One of our primary findings was that the acute ingestion of low caffeine doses (~1.3 to 3 mg·kg^−1^) improved aerobic time-trial outcomes (SMD = −0.27, 95% CI = −0.44 to −0.11). Importantly, when analyzing the raw time-trial outcomes reported across the eligible studies, these effects corresponded to a mean reduction in completion time of approximately 2.14% across the included tests. Furthermore, we observed that this performance enhancement was consistently identified with the use of moderate caffeine dosages (SMD = −0.52, 95% CI = −0.77 to −0.28). When considering the raw performance outcomes reported across the included studies, moderate caffeine doses were associated with a mean improvement in completion time of approximately 2.18%. Notably, although the standardized effect size associated with moderate caffeine doses was greater than the small effect observed with low caffeine doses, standardized mean differences are influenced not only by the magnitude of performance changes but also by variability measures and study precision. Therefore, larger SMD values do not necessarily indicate proportionally greater real-world percentage improvements in exercise performance. Additionally, sensitivity analyses indicated that the difference between low and moderate caffeine doses was partially influenced by the data representation of the studies conducted by Guest et al. (2020) [24] and Hodgson et al. (2013) [51], highlighting the need for careful interpretation regarding the current state of the literature (for further details, see Supplementary Figure S3). Regardless, our findings align with the expected exercise improvements (2–4%) reported by major international organizations [5].

In part, our results corroborate the average effect size reported for acute caffeine ingestion in the meta-analysis conducted by Chen and colleagues (2024) [11], reinforcing that pre-exercise caffeine consumption appears to enhance generalized aerobic time-trial outcomes (e.g., running, cycling, swimming) in a manner comparable to its effect on cycling specifically (moderate effect size reported: 0.5 vs. 0.52 observed in the present analysis). In contrast to the earlier meta-analysis [11], our study is the first to demonstrate that low caffeine doses may improve aerobic exercise outcomes by significantly reducing total completion time during endurance-based exercise tests. This discrepancy may be partially explained by the inclusion of rowing and/or running performance tests in our review, which accounted for 37.5% of all studies using low caffeine doses, whereas Chen et al. (2024) [11] evaluated only cycling performance, which inherently limited the pool of eligible clinical trials.

There is substantial evidence that low caffeine doses (0.5–3 mg·kg^−1^) exert central nervous system (CNS) effects capable of increasing alertness, vigilance, and attention, as well as reducing reaction time and enhancing cognitive focus in humans [75]. Even in studies where caffeine dosage was not standardized relative to body mass, time-trial performance tests have demonstrated that the use of low doses of caffeine (100–200 mg) can improve performance and reduce ratings of perceived exertion [76,77]. Although the results of our meta-analysis reinforce that the ergogenic effects of caffeine on aerobic time-trial performance occur with low doses and increase with the use of moderate doses, it is important to highlight that this dose–benefit pattern was not consistently observed across all studies that examined more than one caffeine dosage. Two studies found no dose-dependent improvements in performance [42,66], whereas three studies [24,27,28] reported greater performance gains with higher caffeine doses.

The substantial interindividual variability observed in the effectiveness of different caffeine doses may be partially explained by genetic polymorphisms affecting caffeine metabolism, particularly within the *CYP1A2* gene, as well as by the relatively small sample sizes frequently observed in caffeine-related exercise studies [5,8]. More specifically, recent evidence from a systematic review and meta-analysis investigating *CYP1A2* genotype and exercise performance suggests that *CYP1A2*-related variants may represent one of the primary genetic factors influencing ergogenic responsiveness to caffeine supplementation, with individuals carrying the AA and AC genotypes more frequently demonstrating ergogenic benefits following caffeine ingestion [78]. In contrast, individuals carrying the CC genotype, commonly classified as slower caffeine metabolizers, may exhibit attenuated or less consistent performance responses to caffeine supplementation across different exercise modalities [23,78]. In parallel, emerging neurophysiological evidence also suggests that additional receptor-related polymorphisms, including adenosine- and serotonin-related pathways, may contribute to variability in caffeine responsiveness [24,79,80]; however, the current evidence supporting these mechanisms in humans remains limited and requires further investigation with regard to perceived exertion, aerobic performance, and interindividual sensitivity to acute stimulant use.

Finally, it should be noted that no eligible clinical trials were identified using high caffeine doses (>6 mg·kg^−1^) before aerobic time-trial performance tests. Although some investigations administering high caffeine doses have been published, including the studies by Graham and Spriet (1991, 1995) [81,82], Pallarés et al. (2013) [83], and Wilk et al. (2019) [84], these investigations employed non-time-trial exercise protocols and/or outcome measures not exclusively based on time-to-completion performance. Additionally, potentially relevant aerobic time-trial studies conducted by Bruce et al. (2000) [85] and Cohen et al. (1996) [86] could not be quantitatively synthesized because the mean and standard deviation values required for meta-analysis were not available. Interestingly, a recent meta-analysis investigating cycling performance quantitatively synthesized only studies administering caffeine doses up to 6 mg·kg^−1^ [11].

Collectively, these findings highlight an important gap in the current literature regarding the effects of high caffeine doses on ecologically valid aerobic performance outcomes. From this perspective, it is essential that future clinical trials examine the risk–benefit profile of high caffeine doses (>6 mg·kg^−1^), both in terms of aerobic time-trial performance and in relation to the potential adverse effects commonly associated with high caffeine intake, such as anxiety, heart palpitations, headaches, insomnia, and gastrointestinal disorders [87].

## 5. Conclusions

This systematic review and meta-analysis demonstrated that the pre-exercise use of low caffeine doses (1.3–3 mg·kg^−1^) can enhance generalized aerobic time-trial performance (Mean Effect Size: 0.27; *p* = 0.001). In addition, the use of moderate caffeine doses (4–6 mg·kg^−1^) appears to promote a more consistent ergogenic effect, reducing total completion time in aerobic time-trial tests (Mean Effect Size: −0.52; *p* < 0.00001). Exploratory subgroup analyses also demonstrated significant ergogenic effects of moderate caffeine doses in both trained and highly trained individuals. Although a dose–response effect was observed, studies employing moderate caffeine doses displayed high heterogeneity (*I*^2^ = 73%) and a wider range of effect sizes (−0.77 to −0.28). This variability may be partly attributable to the limited number of published studies using moderate doses, as well as the substantial statistical influence of one eligible study. Finally, no previously published studies investigating the use of high caffeine doses (>6 mg·kg^−1^) in aerobic time-trial performance were deemed eligible. This finding underscores the lack of high-quality research examining the effects of high caffeine dosages.

From a practical perspective, the present findings suggest that low and moderate caffeine doses may represent effective supplementation strategies for improving aerobic time-trial performance, indicating that caffeine may exert ergogenic effects within low-to-moderate dosage ranges in aerobic time-trial contexts. Future investigations should further explore the effects of different caffeine doses according to individual sensitivity and genetic characteristics of endurance athletes, particularly involving *CYP1A2* (associated with caffeine metabolism) and other genetic factors potentially involved in adenosine- and serotonin-related neurophysiological pathways underlying caffeine responsiveness. In addition, we highlight the importance of future investigations exploring the performance effects of high caffeine doses (6.1–9 mg·kg^−1^) on aerobic performance, metabolism, and the incidence of adverse effects during aerobic time-trial performance tests. Taken together, these future research directions may help clarify the dose–response effects of caffeine supplementation on aerobic exercise performance and support the development of more personalized nutritional strategies based on individual physiological and genetic characteristics.

## Data Availability

No new data were created or analyzed in this study. Data sharing is not applicable to this article.

## Supplementary Materials

The following supporting information can be downloaded at: https://www.mdpi.com/article/10.3390/nu18121989/s1, Figure S1. Risk of bias summary: review authors’ judgements about each risk of bias item for each included study. Figure S2. Exploratory subgroup analysis according to aerobic training status among studies administering moderate caffeine doses (4–6 mg·kg^−1^). Figure S3. Forest plot for the effect of interventions using moderate doses of caffeine (4 to 6 mg·kg^−1^) and placebo control on performance in aerobic time trials, without the presence of the pooled analysis of studies that fell outside the 95% Confidence Interval (95% CI) of the mean result reported among studies that investigated the administration of moderate doses of caffeine [24,51].
