# Supplementary material for: Dose-Response Effect of Oral Caffeine Use on Aerobic Exercise Performance: A Systematic Review and Meta-Analysis
Source: Nutrients. 2026 Jun 19;18(12):1989. doi: 10.3390/nu18121989 (PMC13305852; doi:10.3390/nu18121989)
Supplement: Supplementary file 1 [file nutrients-18-01989-s001.zip › Supplementary Figure S3.pdf]

Study or Subgroup	Experimental (caffeine) Mean	SD	Total	Control (placebo) Mean	SD	Total	Weight	Std. Mean Difference IV, Random, 95% CI
ACKER-HEWITT et al. (2012)	2,616	294	10	2,652	270	10	2.2%	-0.12 [-1.00, 0.76]
AL-NAWAISEH et al. (2020)	1,074.9	119.7	11	1,053.8	86.8	11	2.4%	0.19 [-0.64, 1.03]
ASTORINO et al. (2011) Physically Active	1,111.8	36.6	8	1,122.6	40.8	8	1.7%	-0.26 [-1.25, 0.72]
ASTORINO et al. (2011) Very Trained	1,024.2	59.4	8	1,041	58.8	8	1.7%	-0.27 [-1.25, 0.72]
ASTORINO et al. (2012a)	1,062	60	10	1,092	66	10	2.1%	-0.46 [-1.35, 0.44]
ASTORINO et al. (2012b)	1,018.8	57.6	9	1,035	57.6	9	1.9%	-0.27 [-1.20, 0.66]
BELL et al. (2002)	2,760	168	12	2,808	192	12	2.6%	-0.26 [-1.06, 0.55]
BLOOMER et al. (2011)	3,120	112.8	12	3,153	117.6	12	2.6%	-0.28 [-1.08, 0.53]
BORBA et al. (2019)	456.6	61.2	13	458.4	48	13	2.8%	-0.03 [-0.80, 0.74]
CONWAY et al. (2003)	1,428	168	8	1,698	186	8	1.3%	-1.44 [-2.58, -0.30]
COUTO et al. (2022)	368.2	14.9	9	375.1	14.5	9	1.9%	-0.45 [-1.39, 0.49]
COX et al. (2002)	1,690.8	40	12	1,750.8	44	12	2.0%	-1.38 [-2.28, -0.47]
DESBROW et al. (2012) 6mg of caffeine.kg−1	3,791	281	16	3,902	340	16	3.4%	-0.35 [-1.05, 0.35]
FELIPPE et al. (2018)	396	5	11	403	6	11	2.0%	-1.22 [-2.14, -0.29]
FERREIRA VIANA et al. (2020)	350	14.6	9	357	13.2	9	1.9%	-0.48 [-1.42, 0.46]
FRANCO-ALVARENGA et al. (2019)	1,944	72	12	1,980	72	12	2.5%	-0.48 [-1.30, 0.33]
GLAISTER et al. (2015)	2,077.2	75.6	14	2,122.2	102	14	3.0%	-0.49 [-1.24, 0.27]
GLAISTER et al. (2021)	1,782	108	40	1,848	138	40	8.4%	-0.53 [-0.97, -0.08]
GONÇALVES et al. (2017)	1,795.2	130.8	40	1,848.6	156	40	8.6%	-0.37 [-0.81, 0.07]
GRAHAM-PAULSON et al. (2016)	1,450	86	11	1,476	67	11	2.4%	-0.32 [-1.17, 0.52]
GUEST et al. (2020) 4mg of caffeine.kg−1	1,056	12	100	1,086	12	100		Not estimable
HANSON et al. (2019) 6mg of caffeine.kg−1	3,162	492	10	3,192	480	10	2.2%	-0.06 [-0.94, 0.82]
HODGSON et al. (2013)	2,301	28.8	8	2,403.6	23.4	8		Not estimable
MACINTOSH et al. (1995)	1,258.8	36.4	11	1,281.8	38.2	11	2.3%	-0.59 [-1.45, 0.26]
MORALES et al. (2020)	1,631	90.45	14	1,674	72.88	14	2.9%	-0.51 [-1.26, 0.25]
O´ROURKE et al., (2008) Well Trained	1,047	69	15	1,058	68	15	3.3%	-0.16 [-0.87, 0.56]
O´ROURKE et al. (2008) Recreational Trained	1,286	86	15	1,298	84	15	3.3%	-0.14 [-0.85, 0.58]
POLLOW et al. (2016)	5,010	156	7	5,040	102	7	1.5%	-0.21 [-1.26, 0.84]
POTGIETER et al. (2018)	8,976	1,188	26	9,090	1,116	26	5.7%	-0.10 [-0.64, 0.45]
ROELANDS et al. (2011)	2,262	312	8	2,196	198	8	1.7%	0.24 [-0.75, 1.22]
SANTOS et al. (2013)	409	12	8	419	13	8	1.6%	-0.76 [-1.78, 0.27]
SANTOS et al. (2020) High Performance	365.5	13.1	8	371	11.4	8	1.7%	-0.42 [-1.42, 0.57]
SANTOS et al. (2020) Low Performance	402.5	16.1	8	412.7	15.6	8	1.6%	-0.61 [-1.62, 0.40]
SILVA- CALVACANTE et al. (2013)	404.6	17.1	7	421	12.3	7	1.3%	-1.03 [-2.17, 0.11]
SKINNER et al. (2010) 4mg of caffeine.kg−1	401.1	19.8	10	403.8	21	10	2.2%	-0.13 [-1.00, 0.75]
SKINNER et al. (2010) 6mg of caffeine.kg−1	402.6	21.2	10	403.8	21	10	2.2%	-0.05 [-0.93, 0.82]
SKINNER et al. (2013)	3,475.7	97.7	14	3,546.2	122.8	14	2.9%	-0.62 [-1.38, 0.14]
STADHEIM et al. (2013)	1,981	84	10	2,066	85	10	1.9%	-0.96 [-1.90, -0.03]
TOMAZINI et al. (2022)	369.1	15.2	11	373	14.8	11	2.4%	-0.25 [-1.09, 0.59]
WALKER et al. (2008)	1,626	102	9	1,692	114	9	1.9%	-0.58 [-1.53, 0.37]
**Total (95% CI)**			**476**			**476**	**100.0%**	**-0.39 [-0.52, -0.26]**

Heterogeneity: $Tau^2 = 0.00$; $Chi^2 = 24.31$, df = 37 (P = 0.95); $I^2 = 0\%$

Test for overall effect: Z = 5.91 (P < 0.00001)

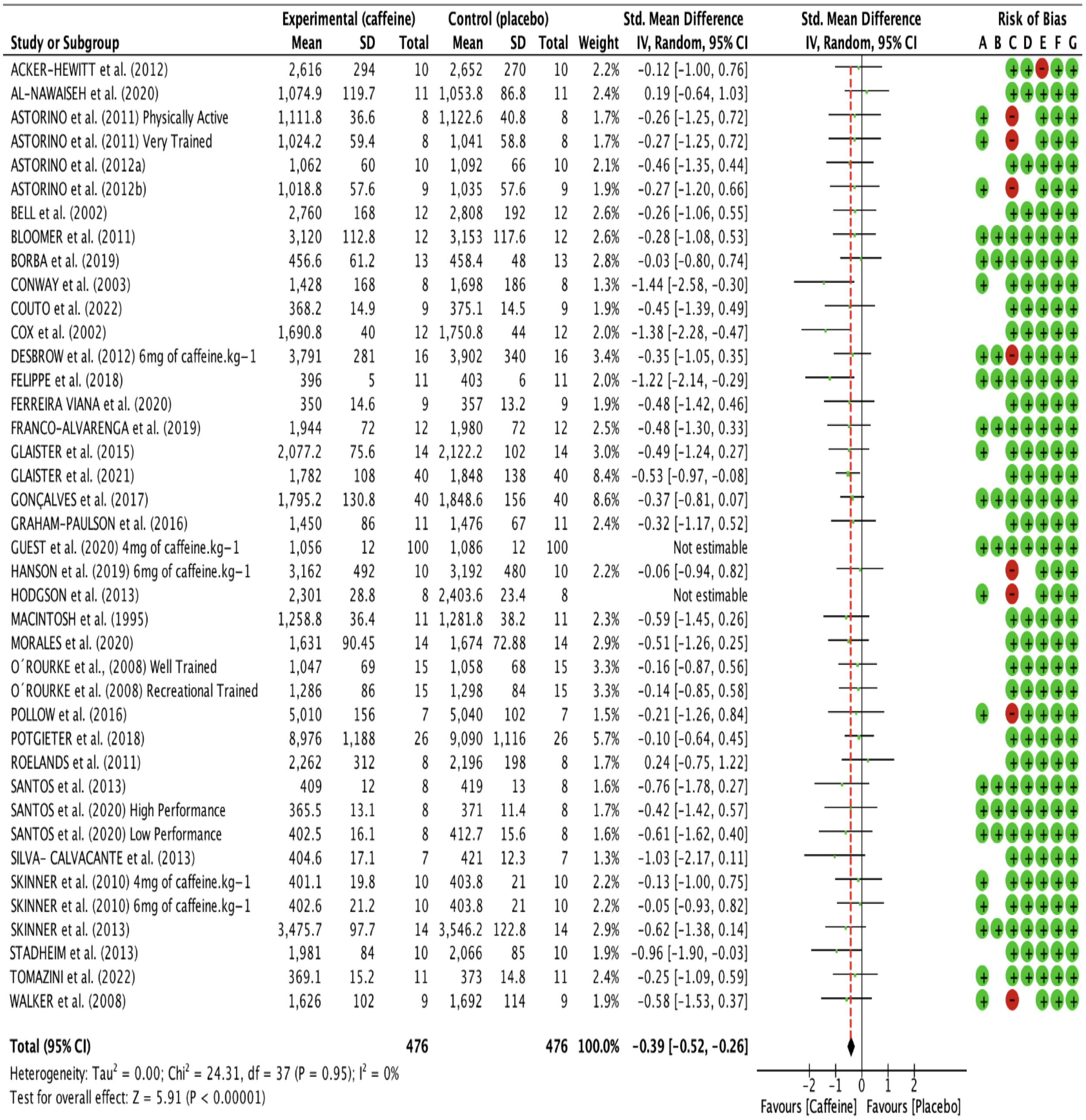

Risk of bias legend

(A) Random sequence generation (selection bias)

(B) Allocation concealment (selection bias)

(C) Blinding of participants and personnel (performance bias)

(D) Blinding of outcome assessment (detection bias)

(E) Incomplete outcome data (attrition bias)

(F) Selective reporting (reporting bias)

(G) Other bias

Supplementary Figure S3. Forest plot for the effect of interventions using moderate doses of caffeine (4 to 6 mg·kg^{-1}) and placebo control on performance in aerobic time trials, without the presence of the pooled analysis of studies that fell outside the 95% Confidence Interval (95% CI) of the mean result reported among studies that investigated the administration of moderate doses of caffeine (GUEST et al. 2020; HODGSON et al. 2013). The analysis of the effects of the data was performed randomly, with the overall mean effect and respective standard deviation represented by a 95% CI. The chi-square (I2) percentage value represents the percentage of heterogeneity among the samples of the studies included in this meta-analysis. All time measurements computed in this meta-analysis were parameterized in seconds, with the mean performance time values for each treatment condition placed in the "Mean" column and their respective standard deviations in the "SD" column. The forest plot scale was set to 3.99 for better comparison with other analyses. Studies included in this analysis: [24,26,28-36,38-40,42,44-51,55-57,59,60,62-64,66,67,70-72].
