# Supplementary material for: Dose-Response Effect of Oral Caffeine Use on Aerobic Exercise Performance: A Systematic Review and Meta-Analysis
Source: Nutrients. 2026 Jun 19;18(12):1989. doi: 10.3390/nu18121989 (PMC13305852; doi:10.3390/nu18121989)
Supplement: Supplementary file 1 [file nutrients-18-01989-s001.zip › Supplementary Figure S1. Risk of bias summary.pdf]

Study	Random sequence generation (selection bias)	Allocation concealment (selection bias)	Blinding of participants and personnel (performance bias)	Blinding of outcome assessment (detection bias)	Incomplete outcome data (attrition bias)	Selective reporting (reporting bias)	Other bias
ACKER-HEWITT et al. (2012)			+	+	−	+	+
AL-NAWAISEH et al. (2020)			+	+	+	+	+
ASTORINO et al. (2011)	+		−		+	+	+
ASTORINO et al. (2012a)			+	+	+	+	+
ASTORINO et al. (2012b)	+		−		+	+	+
BELL et al. (2002)			+	+	+	+	+
BLOOMER et al. (2011)	+	+	+	+	+	+	+
BORBA et al. (2019)	+	+	+	+	+	+	+
BRIDGE et al. (2006)			+	+	+	+	+
CONWAY et al. (2003)	+		+	+	+	+	+
COUTO et al. (2022)			+	+	+	+	+
COX et al. (2002)			+	+	+	+	+
DEAN et al. (2009)			+	+	+	+	+
DESBROW et al. (2009)	+	+	+	+	+	+	+
DESBROW et al. (2012)	+	+	+	+	+	+	+
DUNCAN et al. (2016)			+	+	+	+	+
FELIPPE et al. (2018)	+	+	+	+	+	+	+
FERREIRA VIANA et al. (2020)			+	+	+	+	+
FRANCO-ALVARENGA et al. (2019)	+	+	+	+	+	+	+
GLAISTER et al. (2015)	+		+	+	+	+	+
GLAISTER et al. (2021)			+	+	+	+	+
GONÇALVES et al. (2017)	+	+	+	+	+	+	+
GRAHAM-PAULSON et al. (2016)			+	+	+	+	+
GUEST et al. (2020)	+	+	+	+	+	+	+
HANSON et al. (2019)	+	+	−		+	+	+
HODGSON et al. (2013)	+		−		+	+	+
IRWIN et al. (2011)	+		+	+	+	+	+
KHCHAREM et al. (2021)	+		+	+	+	+	+
KILDING et al. (2012)			+	+	+	+	+
MACINTOSH et al. (1995)			+	+	+	+	+
MORALES et al. (2020)			+	+	+	+	+
O´ROURKE et al. (2008)			+	+	+	+	+
PITCHFORD et al. (2014)			+	+	+	+	+
POLLOW et al. (2016)	+		−	+	+	+	+
POTGIETER et al. (2018)			+	+	+	+	+
QUINLIVAN et al. (2015)			+	+	+	+	+
ROELANDS et al. (2011)			+	+	+	+	+
SANTOS et al. (2013)	+	+	+	+	+	+	+
SANTOS et al. (2020)	+	+	+	+	+	+	+
SCOTT et al. (2015)	+		−		+	+	+
SILVA- CALVACANTE et al. (2013)			+	+	+	+	+
SKINNER et al. (2010)	+		+	+	+	+	+
SKINNER et al. (2013)	+	+	+	+	+	+	+
SKINNER et al. (2019)	+		−	+	+	+	+
SPENCE et al. (2013)	+		+	+	+	+	+
STADHEIM et al. (2013)			+	+	+	+	+
TOMAZINI et al. (2022)	+		+	+	+	+	+
WALKER et al. (2008)	+		−		+	+	+

Supplementary Figure S1. Risk of bias summary: review authors' judgements about each risk of bias item for each included study. Green shading indicates a low risk of bias for the corresponding domain assessed; red shading indicates a high risk of bias for the corresponding domain assessed; absence of shading indicates an unclear risk of bias due to insufficient reported methodological information in the original articles.
