# Supplementary material for: Dose-Response Effect of Oral Caffeine Use on Aerobic Exercise Performance: A Systematic Review and Meta-Analysis
Source: Nutrients. 2026 Jun 19;18(12):1989. doi: 10.3390/nu18121989 (PMC13305852; doi:10.3390/nu18121989)
Supplement: Supplementary file 1 [file nutrients-18-01989-s001.zip › Supplementary Figure S2.pdf]

Study or Subgroup	Caffeine Mean	Caffeine SD	Caffeine Total	Placebo Mean	Placebo SD	Placebo Total	Weight	Std. Mean Difference IV, Random, 95% CI
**1.3.1 Trained Individuals**								
ASTORINO et al. (2011) Physically Active	1,111.8	36.6	8	1,122.6	40.8	8	7.2%	-0.26 [-1.25, 0.72]
FELIPPE et al. (2018)	396	5	11	403	6	11	7.4%	-1.22 [-2.14, -0.29]
GLAISTER et al. (2015)	2,077.2	75.6	14	2,122.2	102	14	8.0%	-0.49 [-1.24, 0.27]
GONÇALVES et al. (2017)	1,795.2	130.8	40	1,848.6	156	40	8.9%	-0.37 [-0.81, 0.07]
GRAHAM-PAULSON et al. (2016)	1,450	86	11	1,476	67	11	7.7%	-0.32 [-1.17, 0.52]
GUEST et al. (2020) 4mg of caffeine.kg−1	1,056	12	100	1,086	12	100	9.1%	-2.49 [-2.86, -2.12]
MORALES et al. (2020)	1,631	90.45	14	1,674	72.88	14	8.0%	-0.51 [-1.26, 0.25]
SANTOS et al. (2020) Low Performance	402.5	16.1	8	412.7	15.6	8	7.1%	-0.61 [-1.62, 0.40]
**Subtotal (95% CI)**			**206**			**206**	**63.3%**	**-0.81 [-1.59, -0.02]**
Heterogeneity: $Tau^2 = 1.11$; $Chi^2 = 75.73$, df = 7 ($P < 0.00001$); $I^2 = 91\%$								
Test for overall effect: Z = 2.02 (P = 0.04)								
**1.3.2 Highly Trained Participants**								
CONWAY et al. (2003)	1,428	168	8	1,698	186	8	6.6%	-1.44 [-2.58, -0.30]
COX et al. (2002)	1,690.8	40	12	1,750.8	44	12	7.5%	-1.38 [-2.28, -0.47]
SKINNER et al. (2013)	3,475.7	97.7	14	3,546.2	122.8	14	8.0%	-0.62 [-1.38, 0.14]
STADHEIM et al. (2013)	1,981	84	10	2,066	85	10	7.3%	-0.96 [-1.90, -0.03]
WALKER et al. (2008)	1,626	102	9	1,692	114	9	7.3%	-0.58 [-1.53, 0.37]
**Subtotal (95% CI)**			**53**			**53**	**36.7%**	**-0.94 [-1.35, -0.53]**
Heterogeneity: $Tau^2 = 0.00$; $Chi^2 = 2.89$, df = 4 (P = 0.58); $I^2 = 0\%$								
Test for overall effect: Z = 4.49 ($P < 0.00001$)								
**Total (95% CI)**			**259**			**259**	**100.0%**	**-0.88 [-1.41, -0.34]**
Heterogeneity: $Tau^2 = 0.78$; $Chi^2 = 79.78$, df = 12 ($P < 0.00001$); $I^2 = 85\%$								
Test for overall effect: Z = 3.23 (P = 0.001)								
Test for subgroup differences: $Chi^2 = 0.09$, df = 1 (P = 0.77), $I^2 = 0\%$								

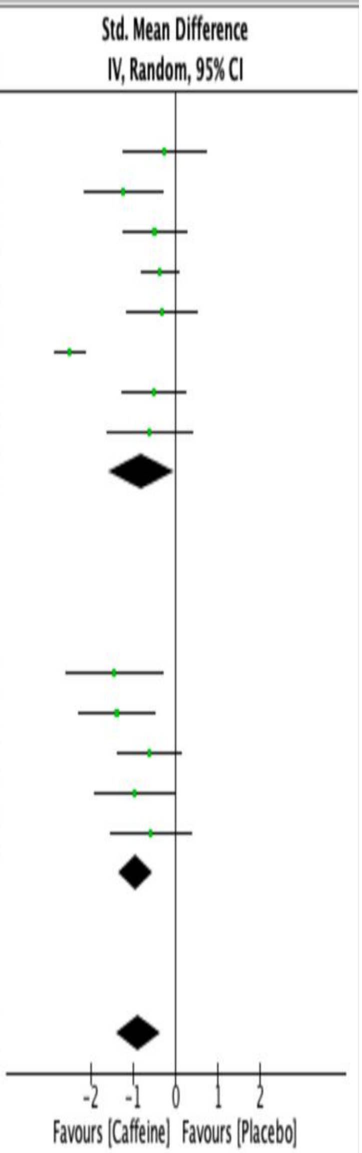

Supplementary Figure S2. Exploratory subgroup analysis according to aerobic training status among studies administering moderate caffeine doses (4–6 mg·kg^{-1}). Participants presenting VO_2max/VO_2peak values between approximately 40–60 mL·kg^{-1}·min^{-1} were categorized as trained individuals, whereas participants with VO_2max/VO_2peak values above 60 mL·kg^{-1}·min^{-1} were categorized as highly trained individuals. Studies that did not report VO_2max/VO_2peak values were excluded from this exploratory subgroup analysis. Additionally, studies presenting substantial within-sample variability in aerobic capacity, including participants distributed across both training-status categories, were not included in the subgroup categorization in order to improve the physiological interpretability of the analyses. All time measurements computed in this meta-analysis were parameterized in seconds, with the mean performance time values for each treatment condition placed in the “Mean” column and their respective standard deviations in the “SD” column. The forest plot scale was set to 3.99 for better comparison with other analyses. Studies included in the trained subgroup: [24,31,44,47,49,50,56,63]. Studies included in the highly trained subgroup: [38,40,67,70,72].
